# Methods in obtaining split-thickness skin grafts from skin reduction surgery specimens

**DOI:** 10.1186/s40064-016-2330-2

**Published:** 2016-05-25

**Authors:** Rebecca E. Bruccoleri, Michael K. Matthew, John T. Schulz

**Affiliations:** Department of Emergency Medicine, Yale-New Haven Medical Center, 464 Congress Avenue, Ste. 260, New Haven, CT 06519 USA; Department of Plastic Surgery, Dartmouth Hitchcock Medical Center, One Medical Center Dr., Lebanon, NH 03750 USA; Sumner Redstone Burn Center, Massachusetts General Hospital, 55 Fruit Street, Boston, MA 02115 USA

**Keywords:** Living donor, Skin graft, Abdominoplasty, Dermatome

## Abstract

**Background:**

To devise a method for obtaining bacterial culture-negative split-thickness skin grafts from specimens removed from living donors undergoing skin reduction surgery.

**Methods:**

Specimens were obtained from patients undergoing abdominal skin reduction surgery in inpatient and outpatient surgical settings. Skin specimens were cleaned in a method adapted from the former Yale Skin Bank’s methods. The specimens were attached to the autoclave container for the dermatome using towel clips or sutures to provide tension. Normal saline clysis was injected subdermally and a Padgett Electric Dermatome was used to obtain skin grafts. These were then photographed and discarded.

**Results:**

Eight specimens were obtained from seven women and one man. The mean age was 46.6 years and mean weight at time of surgery was 87.7 kg. Bacterial cultures obtained from all specimens were negative. All procured grafts were transparent, with visible dermis, suggesting that they could be used in a clinical setting.

**Conclusion:**

Bacterial culture-negative split-thickness skin grafts can be obtained from skin reduction surgery specimens, offering a potential source of split-thickness allograft during regional or national shortages.

## Background

First reported in 1872 by Reverdin, split-thickness skin allograft did not become widely used for covering large burn or wound surfaces until James Barrett Brown published his work in 1942 and 1953 (Hansbrough 1992; Brown and McDowell 1942; Brown et al. 1953). Since that time, split-thickness allograft has become a mainstay for effective temporary wound coverage (Jackson 1954; Macmillan 1962). Although currently skin banks only accept split-thickness skin grafts from cadaveric donors, the idea of using surgically discarded skin as a potential allograft source was described by Brown et al. (1953). Jackson described using allograft from living donors, and Swartz et al. (1975) described a method for procuring and using split-thickness skin from panniculectomy specimens, although they did not report skin culture results or quality of the grafts obtained (Jackson 1954; Swartz et al. 1975).

According to the American Society of Plastic Surgeons, the number of patients undergoing abdominoplasties in 2013 was 111,986 (American Society of Plastic Surgeons 2013). In addition, there were 41,998 procedures after massive weight loss which included 16,602 abdominoplasties (American Society of Plastic Surgeons 2013). The goal of this project was to devise a technical method for obtaining potentially usable split-thickness grafts from specimens removed during skin reduction surgeries after massive weight loss. While not currently used, surgically discarded skin is a potential source for allograft that could be used during times of cadaveric allograft shortage. For example, in 2007, the American Association of Tissue Banks (AATB) reported that 2288 requests for split-thickness skin were denied due to a shortage of 1358 square feet of allograft (American Association of Tissue Banks (AATB) 2007). In the face of such a shortage, skin reduction surgery offers a readily available source of allograft, if procurement of the discarded specimens is technically feasible.

In this article, we present a method for procuring split-thickness skin grafts from discarded surgical specimens, demonstrating that such procurement is technically simple and that specimens can be transferred from the operating room (OR) to procurement and prepared aseptically.

## Methods

The study was a prospective convenience sample of patients undergoing abdominoplasty. Because abdominoplasty specimens would be otherwise discarded, the study was approved as a non-human subjects study, requiring no patient consent, by the Institutional Review Board (Human Investigation Committee Number: 0808004161). Specimens were obtained from two clinical sites: one inpatient hospital and one outpatient medical center. All specimens were of excess skin that was removed electively from patients after massive weight loss. Specimens from patients with an open wound or infection within the resection margins were excluded.

The age, gender, time from bariatric surgery or commencement of weight loss to surgery, amount of weight loss, and site of skin reduction of the patients were recorded. Skin specimens were collected at the time of surgery and procurement of skin grafts occurred within 24 h of collection. If the skin grafts were not immediately procured, the specimens were placed in a refrigerator. Skin grafts were procured either in a laboratory or a back table in the operating room. For grafts procured in the laboratory, specimens were prepped in accordance with the former Yale Skin Bank’s methods. For grafts procured in the operating room, skin specimens were prepared per the surgeon’s methods for the surgery and no additional sterilization of the specimen was performed before skin cultures were taken.

The sterilization procedure for abdominoplasty specimens procured in the laboratory was as follows: the specimen was rinsed of any gross debris with tap water. The specimen was shaved of any excess hair if necessary. The specimen was then scrubbed with a povidone iodine scrub brush, wiped with alcohol, wiped with povidone iodine and then rinsed with a phosphate buffered 0.25 % sodium hypochlorite solute on with pH between 7 and 8 (determined by pH indicator paper or a pH meter). In two cases 3M™ Remover Lotion (3M™, St. Paul, MN) for 3M™ DuraPrep™ Surgical Solution (3M™, St. Paul, MN) was applied before the iodine scrub brush was used. Then a sterile culture swab was wiped over the surface of the specimen in multiple sites and aerobic cultures were performed by the hospital microbiology laboratory.

In the case of the skin grafts procured in the operating room, the scrub technologist obtained cultures for aerobic culture testing before the specimen was transferred to the back table. Then 3M™ Remover Lotion was applied, the skin was shaved of excess hair and the specimen was wiped again with 3M™ Remover Lotion.

Once the cultures were obtained, the skin was secured with either maxon sutures or towel clips to a sterile autoclave container for the Padgett Electric Dermatome turned upside down. The autoclave container was used to mount the skin specimens since it had holes that were approximately 0.5 cm apart that sutures or towel clips could be threaded through to apply tension. Given that the underlying subcutaneous tissue was uneven and therefore the surface of each specimen was sloped, clysis was performed on all specimens and was necessary to provide a flat surface for which to use the dermatome. The skin and Padgett Electric Dermatome were then wiped with mineral oil and specimens in the nominal depth range of 15–20 thousandths of an inch were procured. The specimens were then spread out on the remaining blades of the dermatome and in some cases with pieces of packaging material underneath to show that the specimen was thin enough such that words were legible underneath the specimen. The specimens were then photographed with a digital camera (Canon^®^ PowerShot A560, Canon USA, Inc., Melville, NY) and discarded. The approximate amount of time to prepare a specimen after cultures were obtained was about 40 min for suturing and 15 min for towel clips.

Analysis of the demographic data was performed with Microsoft Excel 2007 edition (Redmond, Washington). In addition, pixel analysis of the photographs to determine the fraction of the specimen surface procured was performed using ImageJ 1.44p (Wayne Rasband, National Institutes of Health). This was performed by outlining the whole specimen and each graft site and then measuring the areas in pixels. The areas of each graft sites were summed and this was divided by the area of the whole graft to obtain the fraction using Microsoft Excel 2007 edition (Redmond, Washington).

## Results

We obtained abdominoplasty specimens from seven women and one man all of whom had successfully achieved weight loss through diet or surgery who later required reconstructive surgery. The patients ranged in age from 27 to 63 years. The mean age was 46.6 years and mean weight at time of surgery was 87.7 kg. One patient had liposuction performed before the abdominoplasty. Bacterial cultures obtained from all specimens were negative. See Table 1 for characteristics of patients and culture results.

**Table 1:** Characteristics of patients

Patient no.	Weight (kg)	Weight loss (kg)	Date of weight loss	Age	Gender	Site	Culture results	Method of weight loss
1	94.0	Approx. 45.4	January 2006	62	Female	Abdomen	NG	Laparoscopic GB
2	70.3	Unknown	7 years ago	63	Female	Abdomen	NG	Gastic Banding
3	108.9	68.0	1 year	27	Male	Abdomen	NG	No GB
4	79.4	Unknown	Exact time unknown but approx. 25 years	45	Female	Abdomen	NG	No GB
5	108.9	62.1	February 2007	55	Female	Abdomen	NG	Laparoscopic GB
6	86.1	45.4	August 2008	34	Female	Abdomen	NG	GB
7	77.1	40.8	2008	29	Female	Abdomen	NG	Laparoscopic GB—1800 cc lipoaspirate recovered before abdominoplasty performed
8	77.1	59.0	Unknown	58	Female	Abdomen	NG	GB

*NG* no growth, *GB* gastric bypass, *Approx*. approximately

**Fig. 1:**
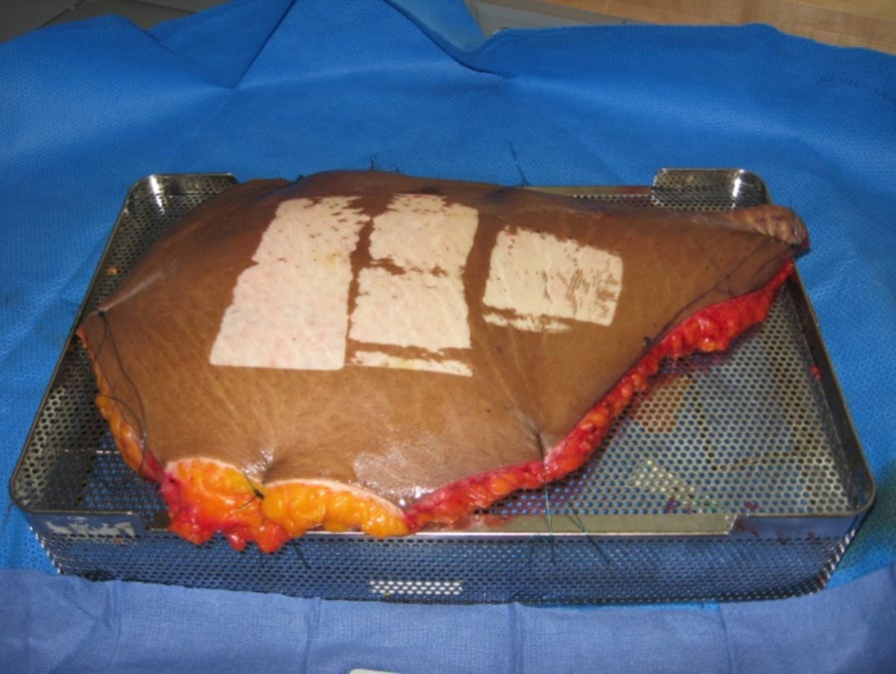
Skin bed of skin graft specimen after graft acquisition

**Fig. 2:**
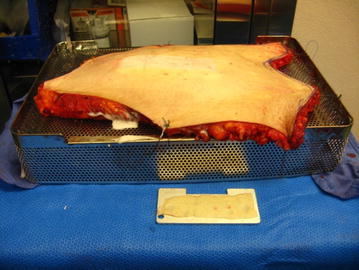
Skin bed of skin graft specimen after graft acquisition along with the graft obtained

All procured grafts were transparent, with visible dermis, suggesting that they could be used in a clinical setting and this was independent of age, gender, amount of weight loss, time from bariatric surgery or commencement of weight loss to surgery. The fraction of easily obtainable split-thickness skin obtained from each specimen varied from 0.12 to 0.34 and some specimens were not assessable (see Table 2). Secondary to time constraints for use of the laboratory and the OR, not all possible grafts were obtained. Examples of apparatus and skin graft beds after procurement can be seen in Figs. 1 and 2. Figure 2 shows a skin graft obtained.

**Table 2:** Fraction of area of skin grafts obtained to area of specimen

Specimen number	Number of grafts obtained	Fraction area grafts obtained	Comments
1	4	0.34	
2	4	NA	Unable to obtain secondary to photograph quality
3	1	NA	More grafts were not obtained secondary to time constraints in OR
4	7	0.25	
5	5	NA	Unable to obtain secondary to photograph quality
6	6	0.27	
7	2	0.12	
8	4	0.29

## Discussion

In this small study we have demonstrated that it is technically possible to aseptically obtain skin grafts from abdominoplasty specimens and that the procedure can be performed immediately after specimen excision in the operating room or the following day in a laboratory. This is contrast to procurement of cadaveric skin grafts which involves personnel being on-call to obtain skin grafts during off hours. Given that abdominoplasty specimens are plentiful, we have demonstrated a method of using this skin to create split-thickness skin grafts which has the potential to supply the need for unfilled requests for skin grafts. From the 2007 AATB survey, tissue banks distributed 314,832 packages of skin. The total square feet reported was 29,908, however 2 of the 24 skin banks did not report their distribution in square feet. Nonetheless, 2288 requests and 1358 square feet were unfilled in 2007 (American Association of Tissue Banks (AATB) 2007). The increased ease and efficiency of harvesting grafts from life donors undergoing skin reduction surgery may be a way to increase the supply while decreasing cost to the system. In addition, this method could also be used by other countries that are not able to fill requests for skin grafts. For example, a need for tissue bank expansion involving skin grafts has been described in China. Given the skin shortage in China, Wang et al. performed a study to begin to evaluate the possibility of hepatitis B transmission through using skin donors who were hepatitis B positive (Wang et al. 2015).

We developed our method using tools that were readily available, although with a relative low yield per specimen. However, other possibilities for obtaining skin grafts exist. One possibility is to attach the specimen to a cylinder and then obtain specimens perpendicular to the axis of the specimen. An additional step of trimming the subcutaneous tissue prior to obtain specimens would likely increase yield. One of the main difficulties in obtaining a specimen was lack of stabilization of the autoclave container to the table. One solution for this problem is to have a sterile vice to attach the autoclave container to the table. In the future, a commercially available kit could be designed to obtain these specimens quickly which would allow for tissue banks to utilize this currently unused resource.

Limitations of this study are small sample size, the fact we did not test preservation methods on these grafts, the majority of specimens were from female subjects, and that the maximum amount of skin grafts were not obtained from every specimen. A more practical concern is that skin banks do not use skin from living donors given concern for transmissible infections. The Centers for Disease Control and Prevention Morbidity and Mortality Weekly Report from May 20, 1994 states: “For semen donations and, when possible, for tissue donations from living donors, the collection should be placed in frozen quarantine and the donor retested for antibodies to HIV-1 and HIV-2 after 6 months (Rogers et al. 1994).” The 6 month quarantine is also stated in the Code of Federal Regulations in 2009 for anonymous donors of semen (Code of Federal Regulations 2009). The skin grafts could be potentially kept frozen for 6 months to allow for retesting of the donor before transplantation. Retesting may decrease the number of donors willing to undergo repeat testing, but given the large numbers of potential donors even a smaller fraction would yield a significant amount of tissue. While the skin grafts in this study were not preserved, they were qualitatively not different from cadaveric grafts and would have been preserved in the same manner. With further work to show that preservation maintains specimen quality along with reliable repeat laboratory testing, this method of obtaining skin grafts could potentially supply skin grafts to the vulnerable and critically ill patient population of burn and trauma patients.

## Conclusion

Although this is a small study, potentially viable skin grafts that meet microbiological criteria for transplant were obtained from all specimens. Therefore, we conclude that it is possible to obtain skin grafts from tissue from living donors obtained after skin reduction surgery.
